# Physical Characters and Morbid Proclivities

**Published:** 1904-04-23

**Authors:** 


### PHYSICAL CHARACTERS AND MORBID PROCLIVITIES.

In the recently-issued volume of *St. Bartholomew's Hospital Reports*, is a paper by Dr. F. C. Shrubsall, in which an attempt is made to substantiate a correlation between racial types and diseases. Although, as the author himself points out, the relatively small number of observations recorded is not sufficient to completely demonstrate the conclusions arrived at, yet they are in some instances very suggestive and certainly appear to indicate that there are fruitful fields open to investigation along the lines that he lays down.

At the outset of the inquiry there are two difficulties to be met. In the first place, the political and ethnic entities, nation and race, are not conterminous, and any conclusions that may be derived from statistics of national disease are thereby vitiated. Secondly there is the inconstant element of social environment, which naturally modifies the liability of any particular subject to disease. In spite of these two sources of fallacy the author is able to arrive at certain fairly definite conclusions which are well deserving of consideration. The bulk of his observations were made on patients at St. Bartholomew's Hospital and the Brompton Hospital, and the physical characters investigated as of special ethnological interest were :—(1) Stature, (2) colour of hair and eyes, (3) head form. With regard to the last mentioned, Dr. Shrubsall notes that there is in the British Isles an almost perfect uniformity in cranial type, a phenomenon which is in marked contrast to that which obtains on the continent of Europe; moreover, on account of the somewhat lengthy nature of the procedure involved and the necessary co-operation of the patient, the observations under this section were not nearly so numerous as those relating to the other characteristics. Because of the necessary disarrangement of the hair, and the passive resistance thus aroused, no measurements were made in the case of women. For these reasons the investigation of cranial form did not lead to any material result. But with regard to stature and complexion, Dr. Shrubsall is able to conclude that there are indications that certain physical types appear with greater frequency among patients from certain diseases than among the general population. Thus blonde traits are associated with acute rheumatism, heart disease, tonsillitis, and osteo-arthritis, while nervous affections, pulmonary tuberculosis, and malignant disease show a predilection for individuals with dark complexions, while there are more doubtful indications of a correlation between tall stature and the former and short stature and the latter groups of illness. Further, the geographical distribution of diseases in the British Isles tends to show a relation between the frequency of physical types and the rate of mortality.

